# Exploring the associations between involuntary treatment and gender in a portuguese acute psychiatric unit

**DOI:** 10.1192/j.eurpsy.2024.644

**Published:** 2024-08-27

**Authors:** A. F. Silva, R. M. Lopes, V. S. Melo, C. A. Rodrigues, P. M. Coelho, F. M. A. Santos, I. S. Fernandes, L. P. Delgado

**Affiliations:** ^1^Psychiatry and Mental Health Department, Centro Hospitalar Médio Tejo, Tomar, Portugal

## Abstract

**Introduction:**

Involuntary admission rates differ between gender across various countries. In several European Union countries, men are more frequently involuntarily admitted, while an opposite trend, associating women with involuntary care, has been observed in countries like Switzerland, Brazil, and China.

**Objectives:**

Considering the contradictory evidence about gender and involuntary care in the literature, we aim to analyze the gender patterns of involuntary care in Centro Hospitalar Médio Tejo’s Psychiatric Acute Unit, exploring the gender differences in diagnosis among involuntary patients.

**Methods:**

We stored and analyzed the data using Microsoft Excel and IBM SPSS Statistics. We studied psychiatry admissions at Centro Hospitalar Médio Tejo, Portugal over 2 years. The Acute Psychiatric Unit, located within a general hospital, has 24 beds, and offers acute mental healthcare services to adults aged 18 and above, serving a coverage area of approximately 251,000 residents. As part of our data collection process for all admissions to the Acute Psychiatry Unit, we recorded information such as gender, age, diagnosis at discharge, treatment type (voluntary or involuntary), and length of stay.

**Results:**

From January 1, 2021, to December 31, 2022, there were 686 psychiatry admissions at Centro Hospitalar Médio Tejo, of which 125 (18,2%) were involuntary. The admission rates were approximately 136.6 per 100,000 people annually, with 24.9 being involuntary admissions per 100,000 people annually. In our analysis of involuntary admissions, women had a lower rate of such admissions, making up 6.4%, while men had a higher rate at 11.8%. No other gender identity was mentioned. Schizophrenia-related disorders were the primary cause for involuntary admissions for both genders, with 67.9% for men and 50% for women. Mood disorders were the second most common reason for involuntary admission, accounting for around 40.9% of cases for women and a significantly lower 16% for men. Involuntarily hospitalized patients exhibited longer lengths of stay independently of the gender. Men hospitalized involuntarily tended to be younger, whereas for women, involuntary hospitalizations were associated with older ages.

**Conclusions:**

In conclusion, our study reveals gender differences in psychiatric involuntary admissions, with more men being involuntarily admitted than women. Schizophrenia group disorders were the most common diagnoses among male and female involuntary patients. Furthermore, all hospitalized women exhibited a higher prevalence of mood disorders, a trend that was more pronounced among those admitted involuntarily. These gender trends match the overall patterns seen in the epidemiology of schizophrenia and mood disorders. Additionally, women with schizophrenia generally exhibit better social functioning than men, which may explain the lower needs of involuntary hospitalization.

**Disclosure of Interest:**

None Declared

